# What the online manipulation of linguistic activity can tell us about language and thought

**DOI:** 10.3389/fnbeh.2013.00122

**Published:** 2013-09-17

**Authors:** Lynn K. Perry, Gary Lupyan

**Affiliations:** Department of Psychology, University of Wisconsin-MadisonMadison, WI, USA

**Keywords:** verbal interference, transcranial direct current stimulation (tDCS), language and thought, linguistic relativity, labeling

Questions about the relationship between language and thought have long fascinated psychologists, philosophers, and the general public. One specific question is the extent to which verbal labels causally impact cognitive processes—how does calling an object by a particular name influence the way people categorize it; how does knowing words for mental states influence our reasoning about the minds of others; how does learning and using words like *left* influence our navigation behavior? One way to learn how the words we use to label objects, mental states, or locations affect our thoughts is to increase or decrease the ease with which we can use these words and observe outcomes of these manipulations on “non-linguistic” tasks. For example, if the word *left* enables us to remember which way to turn, preventing its activation might be expected to disrupt navigation. Manipulating the labeling process (and the engagement of language more broadly) is therefore very useful in exploring how language influences cognition. In this paper, we review two methodologies for implementing linguistic manipulations: verbal interference and transcranial direct current stimulation (tDCS), and discuss what we can learn about the role of language in cognitive processes from this line of research.

## Verbal interference

The primary method for manipulating linguistic activity is the use of verbal interference. The logic of verbal interference is well summarized by Winawer et al. (2007) in the context of studying the effects of language on color perception:

“[I]f linguistic processes play an active, online role in perceptual tasks, then a verbal dual task, but not a non-linguistic dual task, should diminish the goluboy/siniy [light blue / dark blue] category advantage found in Russian speakers,” (Winawer et al., 2007, p. 7781).

Winawer et al. reported that Russian speakers appear to perceive a larger perceptual distinction between light and dark blues, consistent with a lexical difference between these categories in Russian. One possibility is that these differences stem from long-term perceptual learning caused by years of distinguishing colors in one language (e.g., Özgen and Davies, 2002). An alternative is that the cross-linguistic perceptual differences arise from online top-down influences of language (e.g., Fonteneau and Davidoff, 2007; Lupyan, 2008). If true, then disrupting these top-down effects in some way may disrupt these online effects of language eliminating the cross-linguistic difference—the pattern observed in the study. It is important to note that for verbal interference to have some effect does not require the involvement of language to be strategic. Rather, the involvement could be one of “the spontaneous but unspoken use of lexical codes,” (Gilbert et al., 2006, p. 489). On this account, previous associations of using the word *blue* to describe the color blue cause the word to become reactivated when the color is seen (Lupyan, 2012a, b). The automatic recruitment of color labels may temporarily warp the perceptual space, producing cross-linguistic differences of the sort observed by Winawer et al.

Using similar logic, verbal interference has been used to argue for a role of language in number concepts (Frank and Barner, 2012), spatial memory (Hermer-Vazquez et al., 1999), categorization (Lupyan, 2009), and theory of mind (Newton and de Villiers, 2007). A similar logic underlies behavioral *up*-regulation of linguistic processes through redundant presentation of labels (e.g., Lupyan et al., 2007) or overt self-directed speech (Lupyan and Swingley, 2012). If internally generated labels support some cognitive or perceptual process, then redundant externally-presented labels can be thought to *up-regulate* the linguistic contribution and verbal interference to *down-regulate* it.

Verbal interference has also been used to examine mechanisms of developmental change. For example, in a task requiring participants to locate an object hidden in the corner of a room, children rely on the room's shape, while adults use one of the walls—painted in a distinct color from the other walls—to find the object (Hermer and Spelke, 1994; cf. Twyman and Newcombe, 2010). While developments in spatial language correlate with developments in using the wall as a landmark, this cannot tell us whether language causally influences spatial memory. In order to determine whether language supports development of spatial memory, Hermer-Vazquez et al. (1999) used a verbal interference paradigm. When adults performed the task while shadowing speech, their performance mirrored that of children's. Thus, in addition to improving our understanding of how language affects cognition in real-time, verbal interference has improved our understanding of how influences of language on cognition develop.

However, the use of verbal interference is not without problems. First, despite being used for many years across many domains, there is at present no working theory of verbal interference. Put bluntly: no one is sure how it works. Its use is vaguely based on the notion that language is a unitary system that can be disrupted, but this assumption is potentially problematic given the degree to which language-related activity is distributed both anatomically (Jung-Beeman, 2005) and functionally, e.g., syntax and semantics, rather than being entirely dissociable systems, tightly interact (MacDonald et al., 1994). Additionally, there is little agreement as to what constitutes verbal interference. Researchers have used numerous tasks, with little theoretical basis for choosing one over another. Tasks falling under the umbrella of verbal interference have included: rehearsing multi-digit numbers for later memory tests (e.g., Gilbert et al., 2006; Lupyan, 2009), repeating the letters “a,b,c” (e.g., Emerson and Miyake, 2003); alternating between naming months and days (e.g., Baddeley et al., 2001), making rhyme judgments (Roberson et al., 2007), answering factual questions such as “What is your name?” (e.g., Hatano et al., 1977), and repeating text, known as speech shadowing (Hermer-Vazquez et al., 1999; Frank and Barner, 2012). These interference tasks differ both in general difficulty and the ease with which performance can be assessed. For example, an interference task with a memory component gives an indication of how well participants rehearsed information—a proxy for how much effort was put into the verbal task—repetition of “a,b,c” does not. Such inconsistencies makes it hard (1) to infer why verbal interference sometimes interferes with primary task performance and sometimes does not and (2) to assess what aspect of language is recruited in the primary task which, when interfered with, disrupts performance.

A final problem with verbal interference is that it requires participants to perform two tasks simultaneously. It is therefore necessary to use a control interference task to determine which changes in primary task performance stem from manipulation to specifically linguistic processes and which stem from having to perform two tasks. Control tasks also vary widely across experiments: from tests of visuospatial memory (e.g., Gilbert et al., 2006; Lupyan, 2009) to foot tapping (Baddeley et al., 2001; Emerson and Miyake, 2003), and rhythm-shadowing (Hermer-Vazquez et al., 1999). Importantly, unless interference tasks are equated in all ways except for their “verbality,” little can be said about the role of language in the primary task. For example, it has been argued that verbal, but not non-verbal interference disrupted performance on a false-belief task (Newton and de Villiers, 2007). However, when the interference tasks were better equated for difficulty, both were similarly disruptive (Dungan and Saxe, 2012). Additionally, because verbal interference uses a dual-task paradigm, participants can exert different amounts of effort into the tasks. Such differential effort is difficult both to measure and control.

Despite its appeal, verbal interference paradigms have clear shortcomings. Below, we outline an alternative way of perturbing linguistic processes that solve *some* of the shortcomings, and advocate for systematic cross-method comparisons of linguistic perturbation methods to more fully inform our understanding of how language augments cognition and perception.

## Transcranial direct current stimulation

One way to avoid some challenges posed by verbal interferences is by manipulating language processing without using secondary tasks through the use of non-invasive brain stimulation. Here, we focus on one such method—tDCS a painless method of regulating cortical excitability through weak electrical current to the scalp—that allows the experimenter to subtly up- and down-regulate neural activity over cortical areas implicated in language processing. For example, using tDCS to up-regulate activity over Wernicke's area (associated with aspects of labeling, particularly comprehension of word meaning; e.g., Price, 2000) is associated with increased ability to map novel words to pictures (Flöel et al., 2008) using it to up-regulate activity over Broca's area (associated with linguistic processes such as speech production; e.g., Gernsbacher and Kaschak, 2003) is associated with increased artificial grammar learning (de Vries et al., 2010).

Similar to using verbal interference, using tDCS to study linguistic influences on cognition assumes that language is a system that can be selectively perturbed. However, tDCS avoids the need to use dual task paradigms. The participant simply performs the main task while undergoing tDCS which, depending on the electrode arrangement, either up- or down-regulates cortical activity in targeted regions. Up-regulating activity is theoretically analogous to behavioral up-regulation through presentation of overt labels (Lupyan, 2008) or behavioral self-directed speech (Lupyan and Swingley, 2012); down-regulating activity is theoretically analogous to verbal interference.

Recently, Lupyan et al. (2012) examined effects of tDCS on non-verbal categorization, showing that down-regulating activity over Broca's area was associated with impairments in the ability to form categories that required selectively representing specific perceptual features to the exclusion of others, e.g., GREEN THINGS, a deficit similar to one shown by individuals performing verbal interference (Lupyan, 2009) or by those with language impairments such as aphasia (Davidoff and Roberson, 2004; Lupyan and Mirman, 2013). In avoiding pitfalls of a dual task design, however, Lupyan and colleagues' tDCS study allows for more definitive conclusions about mechanisms by which language might affect categorization because participants all completed the same task, and between-group differences can be linked to changes in neural activity in a particular cortical region providing at least a foothold for starting to connect behavioral data on the role of language in categorization to particular neural mechanisms.

## Important future considerations

Although tDCS may be well-suited for manipulating linguistic activity, at present there are no direct comparisons of tDCS to verbal interference. It would be useful to know whether domains in which we have seen effects of verbal interference on performance are affected by tDCS over areas associated with language processes and whether domains in which we have *not* previously seen effects are similarly *unaffected*. For example, tDCS can be used to determine if language-related cortical regions shown to be recruited in non-verbal color-judgment tasks (e.g., Ting Siok et al., 2009) are *causally* implicated by showing perturbations with tDCS have behavioral consequences in color-judgment, thereby informing the neural basis of language-augmented perception. tDCS also has the potential to open up additional domains to investigate effects of language on cognition without the error-fraught hunt for perfect control interference tasks.

In sum, we advocate: (1) a more systematic comparison of linguistic perturbation methods, specifically comparing behavioral linguistic perturbations to perturbations utilizing stimulation techniques such as tDCS; (2) a more rigorous comparison of different verbal interference tasks; and (3) a theory that elucidates the mechanisms by which verbal interference actually works. Such an examination will be critical to clarifying the contributions of language to cognition, helping us answer such questions as whether the mechanisms by which language affects perception of color categories are in some broad sense similar to mechanisms by which language affects spatial cognition. Performing the cross-method comparisons we advocate will highlight possible contradictions leading to further theoretical refinement. For example, what would it mean for the underlying cognitive and neural processes if one verbal interference method affects a primary task but another does not?

The extant empirical literature on effects of language on cognition and perception takes us considerably beyond the question as it is often phrased: “Does language affect thought,” (Boroditsky, 2010a). This literature (e.g., Gentner and Goldin-Meadow, 2003; Casasanto, 2008; Boroditsky, 2010b; Lupyan, 2012a, b), while rich in demonstrations, requires a more rigorous investigation of the mechanisms by which learning and using language augment and perhaps fundamentally alter cognition and perception. We believe significant clarity on this important question can be achieved by combining creative uses of linguistic perturbation techniques with theoretical refinement of their mechanisms.
